# The Interactive Effects of eHealth Literacy and Mental Health Literacy on Social Media Addiction and Depression-Anxiety-Stress in Adolescents: Cross-Sectional Study

**DOI:** 10.2196/81741

**Published:** 2025-11-28

**Authors:** Yuting Xu, Zuosong Chen

**Affiliations:** 1 School of Kinesiology and Health Capital University of Physical Education and Sports Beijing, Beijing China; 2 Emerging Interdisciplinary Platform for Medicine and Engineering in Sports (EIPMES) Capital University of Physical Education and Sports Beijing China

**Keywords:** adolescents, mental health literacy, eHealth literacy, mental eHealth literacy, social media addiction, depression-anxiety-stress

## Abstract

**Background:**

Chinese adolescents are navigating a complex digital environment amidst a growing mental health crisis. While health literacy is recognized as protective, previous research has primarily examined eHealth literacy (eHL) and mental health literacy (MHL) in isolation, leaving their synergistic effects and the integrative mechanism of mental eHealth literacy (MeHL) on social media addiction (SMA) and depression-anxiety-stress (DASS) largely unexplored.

**Objective:**

This study aimed to investigate how eHL and MHL work through MeHL to reduce SMA and DASS in adolescents. We specifically tested whether having stronger MHL makes an adolescent's eHL more effective at building their overall MeHL.

**Methods:**

A cross-sectional survey was conducted among 855 high school students (mean age 16.38, SD 0.86 years; 52.5%, 449/855 male) from 5 Chinese provinces, selected via stratified cluster random sampling. Participants completed validated scales for eHL, MHL, MeHL, SMA, and DASS. Data were analyzed using structural equation modeling (SEM) and Hayes' PROCESS macro (model 7) with cluster-robust SEs, controlling for age and gender. The significance of indirect and conditional effects was assessed using bias-corrected bootstrap CIs based on 5000 resamples.

**Results:**

The results revealed distinct pathways. SEM path analysis indicated that both eHL (β=–0.152, 95% CI –0.270 to –0.034; *P*=.01) and MHL (β=–0.261, 95% CI –0.360 to –0.162; *P*<.001) were negatively associated with SMA. In contrast, only MHL demonstrated a significant negative association with DASS (β=–0.590, 95% CI –0.647 to –0.533; *P*<.001), whereas the link from eHL was nonsignificant (β=–0.011, 95% CI –0.102 to 0.080; *P*=.81). Bootstrapping tests confirmed that MeHL served as a significant mediator, with significant indirect paths from both eHL (SMA: β=–0.062, 95% CI –0.125 to –0.010, *P*=.01; DASS: β=–0.037, 95% CI –0.072 to –0.009, *P*=.008) and MHL (SMA: β=–0.045, 95% CI –0.090 to –0.008, *P*=.01; DASS: β=–0.027, 95% CI –0.053 to –0.007, *P*=.007). A key finding was the significant interactive association of eHL and MHL with MeHL (β=0.067, 95% CI 0.010-0.124; *P*<.05). This moderated mediation was supported, as the protective indirect paths from eHL (via MeHL) to both SMA and DASS were significantly stronger at higher levels of MHL.

**Conclusions:**

Unlike previous research that examined them separately, this study shows that eHL and MHL work best when combined. We found that MHL acts like a catalyst; it enhances the value of digital skills, helping teenagers become more competent at finding, understanding, and using online MeHL support effectively. In practice, this means integrated intervention programs that teach digital skills and mental health knowledge together are likely to be much more effective in fighting SMA and reducing distress than teaching either one alone.

## Introduction

### Background

Adolescent mental health is a cornerstone of national well-being and a pivotal focus of China's “Healthy China” initiative. This commitment is demonstrated by consecutive national policies explicitly targeting the enhancement of mental health literacy (MHL). Key actions include the “Healthy China Action (2019-2030),” which sets specific literacy targets, and the “Action Plan for Children and Adolescent Mental Health (2019-2022),” which aimed to boost mental health knowledge among youths [1,2]. This policy momentum has been sustained through recent strategic outlines, underscoring the ongoing priority of safeguarding adolescent psychological well-being [3,4].

Against this policy backdrop, a pressing public health challenge has emerged. National data indicate that 14.8% of adolescents in China face depression risk, with 40% frequently experiencing loneliness [5,6]. This psychological vulnerability coexists with a near-saturated digital environment, characterized by 96.8% internet penetration among minors [7]. Within this context, a pronounced reliance on digital technology is evident, as over one-third of adolescents exhibit psychological dependence on smartphones. Moreover, prolonged social media use is linked to a significantly increased risk of anxiety and depression [8]. This combination of factors means today’s teenagers, who are “digital natives,” face 2 major risks: heightened psychological distress and problematic social media use. Therefore, improving health literacy—particularly in the digital mental health domain (mental eHealth literacy [MeHL])—and mitigating social media addiction (SMA) has emerged as a critical public health imperative.

Enhancing health literacy is widely established as a cornerstone strategy for public health improvement [9,10]. Yet, adolescents often exhibit insufficient health literacy, which manifests as help-seeking dilemmas (eg, “not recognizing illness” or “recognizing but not seeking treatment”) that hinder access to care [11]. This is particularly critical for senior high school students, whose developing literacy can shape lifelong health trajectories. In theory, health literacy is thought not only to directly affect health but also to work by influencing other factors (as a mediator) or by changing the strength of a relationship (as a moderator) [12-15].

However, research has seldom examined the distinct and potentially synergistic roles of its multidimensional facets in the digital context. Specifically, a significant gap remains in understanding the interplay between MHL—focused on psychological cognition, eHealth literacy (eHL)—centered on digital competency, and their integration as MeHL in relation to adolescent SMA and depression-anxiety-stress (DASS). To address this gap, this study aims to investigate the multifaceted effects and underlying mechanisms of this multidimensional health literacy framework, thereby informing targeted mental health interventions.

### Theory and Research Hypotheses

#### Health Literacy, SMA, and DASS

Health literacy, initially proposed by Simonds in 1974, emphasizes improving public health decision-making skills through education [16]. Later, Nutbeam [17] further expanded the concept, proposing a 3-level model of functional, interactive, and critical health literacy, focusing on individuals' ability to acquire health information while emphasizing the ability to assess information and implement health decisions. The World Health Organization defines health literacy as “the ability to access, understand, evaluate, and apply health information and services to promote and maintain good health” [18]. In the “Healthy China Action (2019-2030)” [2], health literacy is viewed as the ability to acquire, process, and understand health-related information, which is essential for maintaining or improving health and quality of life, and is a critical means for improving health outcomes and promoting healthy behaviors. Thus, health literacy is not limited to the acquisition of health knowledge but emphasizes the promotion of overall health through effective health decision-making and behavior change.

As an important branch of health literacy, mental health literacy was first introduced by Jorm et al [19], focusing initially on individuals' ability to recognize, understand, and address mental health issues, while emphasizing the reduction of stigma and the enhancement of help-seeking willingness [20]. As research deepened, the scope expanded to include multiple dimensions such as recognizing mental health problems, understanding causes, and coping strategies [21]. The core feature of MHL focuses on psychological content, emphasizing cognitive processing and attitude transformation toward mental states.

With the widespread penetration of digital technologies, eHL and MeHL have emerged. The former, defined by Norman and Skinner [22], emphasizes the ability to search, evaluate, and apply broad health information through electronic media, highlighting the operational characteristics of media approaches. The latter, as an emerging concept, is defined as “the knowledge, beliefs, and skills to manage mental health through digital tools” [23], which represents an intersection of MHL and eHL within a specific technological context. It is a dual-core integration dimension of “media × psychology.” Although these 3 aspects fall under the health literacy system, they are interconnected but not hierarchically subordinate, and should be understood as parallel and intersecting multidimensional variables [24]. In conclusion, health literacy is a multidimensional, systematic concept that encompasses the acquisition, understanding, judgment, and application of health knowledge and services. It is a learned psychological tendency that is highly interventionable and modifiable [25].

In the current educational context, stress has become a significant psychosocial factor affecting adolescents, with anxiety and depression being the most common mental health disorders [26]. According to data from the China Youth Research Center [7], the detection rate of anxiety among primary and secondary school students is 31.3%, with a depression risk rate of 17.9%. Additionally, 57.4% of high school students report high levels of academic pressure. Many adolescents delay or are unwilling to seek psychological support due to the stigmatization of mental health issues, lack of knowledge about mental health assistance, and insufficient external support [27], reflecting a deficiency in MHL. Adolescent mental health is directly influenced by MHL. Good MHL not only helps to recognize mental disorders [28] but also increases help-seeking willingness and coping ability [29], alleviates negative emotions, and reduces the risk of depression and anxiety [30,31], with a particularly significant improvement observed in adolescents with mild to moderate depression.

eHL, which is the ability to obtain and apply online health information, is also crucial for adolescent mental health. Lower levels of eHL lead to difficulties in understanding information, which in turn affects health management behaviors. Higher levels of eHL are linked to adolescents' ability to acquire effective health resources, higher psychological well-being, and better self-regulation abilities [32]. Furthermore, eHL is negatively correlated with anxiety and depression [33], playing a positive role in relieving psychological stress, enhancing social support, and improving emotional adaptability [34,35].

SMA, as a typical behavioral addiction of the digital age, is characterized by maladaptive dependence on social media platforms and a loss of self-regulation [34]. A representative survey of nearly 6000 adolescents revealed that 4.5% of them are at risk for SMA [36], with prevalence rates generally higher than those in university students and community adults [37]. Existing research primarily explains the mechanisms of SMA from the perspectives of personality traits (eg, emotional stability) and negative emotions (eg, depression and anxiety) [38], with limited attention given to the role of health literacy.

Social cognitive theory (SCT) suggests that the interaction between individuals, behaviors, and environments shapes behavioral patterns, and adolescents' social media use is influenced not only by the media environment but also by their level of health literacy [22]. Research has found that higher MHL is significantly associated with a lower likelihood of SMA and can effectively intervene in maladaptive usage behaviors [39]. eHL has also been shown to be strongly negatively correlated with internet addiction [40]. Adolescents with lower health literacy are more likely to develop internet dependence, using the internet to distract themselves, escape reality, or regulate negative emotions such as loneliness, anxiety, and depression [41]. The information overload and social pressure in the online environment further amplify their negative emotional experiences, particularly in groups with insufficient eHL, which increases the likelihood of feelings of anxiety, isolation, and self-denial [42].

It is crucial to distinguish between the core constructs of “digital literacy” (eg, eHL) and the contextual challenge of “information overload.” Digital literacy refers to an individual's subjective capability to process digital health information, acting as a protective factor. In contrast, information overload represents an objective environmental stressor or risk factor. The core logic of our framework is to investigate how high levels of digital literacy enable adolescents to effectively navigate and mitigate risks within an information-overloaded environment. Additionally, adolescents who use social media for more than 2 hours a day often experience increased DASS and suicidal ideation, highlighting the real threat of addictive behaviors. The higher the level of eHL, the more effectively individuals can use digital resources to regulate emotions and improve behavioral health, demonstrating lower addiction tendencies and higher psychological resilience.

#### The Impact of Health Literacy on SMA and DASS

MeHL is a composite literacy formed by integrating the core elements of MHL and eHL in the context of digital media. It emphasizes an individual's ability to acquire, understand, assess, and apply mental health information using digital tools [43]. Compared to MHL, which focuses on the recognition and management of mental health problems, and eHL, which emphasizes the acquisition and assessment of digital health information, MeHL involves both cognitive processing of psychological content and the technical processing of media information, forming an integrated, rather than subordinate, independent dimension [44].

As an important intermediary pathway through which education levels influence health status, lower MeHL is significantly associated with an increased risk of mental health disorders [45]. Individuals with higher levels of eHL and MHL are better equipped to use digital platforms to access psychological support and intervention resources. This results in stronger MeHL, which enables them to more effectively identify and address psychological risks, thus reducing maladaptive and addictive behaviors associated with social media use [46]. Some studies also suggest that digital literacy is negatively correlated with behavioral addiction, meaning that the higher the digital skills, the better adolescents can avoid excessive social media use [45].

Further research has found that both MHL and eHL have a significant positive effect on predicting health behaviors, and that MHL can play a key moderating role in the process by which eHL influences health behaviors, with its moderating effect reaching 31.1% [47]. Individuals with higher levels of eHL possess the ability to filter information and operate media tools, but without the cognitive support of MHL, they may find it difficult to convert information into effective behavioral intentions. On the other hand, individuals with higher MHL are more likely to proactively identify, understand, and integrate health information in complex digital environments, thereby enhancing their behavioral execution and psychological regulation [48]. This mechanism aligns with the behavioral-driving logic of “perceived benefits” and “self-efficacy” in the health belief model (HBM) [49] and is consistent with the “individual-behavior-environment” triadic interaction pathway proposed by SCT [37].

### This Study

**Figure 1:**
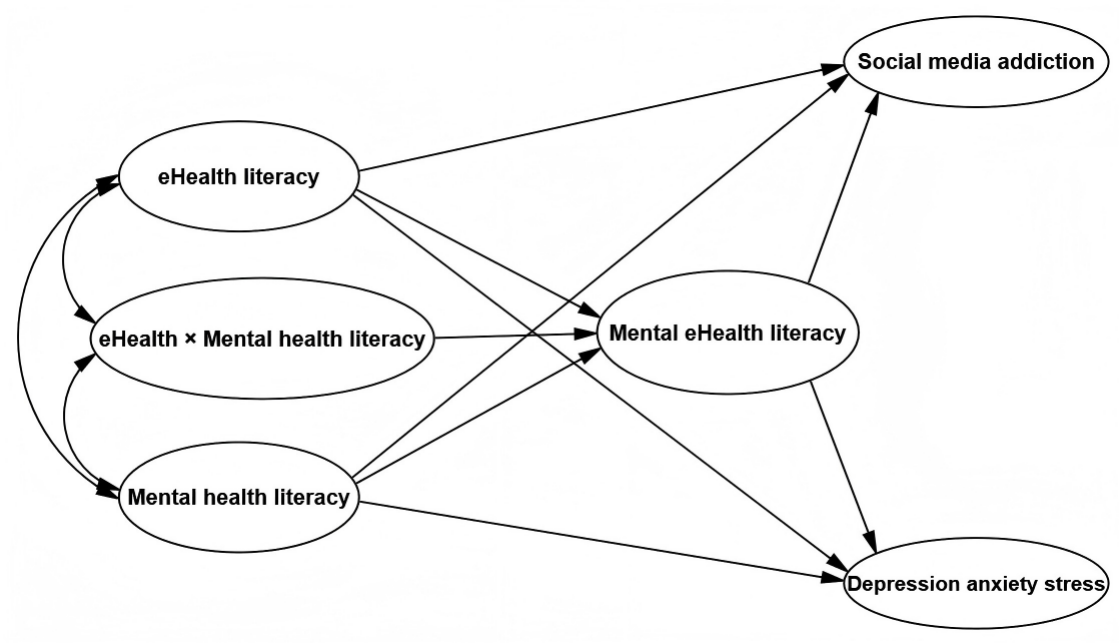
Conceptual framework of the moderated mediation model linking health literacy to social media addiction and depression-anxiety-stress in Chinese adolescents.

Existing research primarily focuses on the independent roles of eHL and MHL, lacking a comprehensive examination of their interactive effects, particularly in the context of SMA. While MeHL is an emerging concept, its role and interactions within the digital environment remain underexplored. To address these gaps through an integrated theoretical lens, this study synthesizes conservation of resources (COR) theory, HBM, and SCT. These theories offer complementary explanations: COR theory provides the motivational foundation by framing health literacy as a key personal resource; HBM elucidates the cognitive decision-making process; and SCT offers the overarching behavioral framework of triadic reciprocity. Guided by this framework, we construct a comprehensive model (Figure 1) to elucidate the joint influence pathways of eHL, MHL, and MeHL on adolescent SMA and DASS. The following hypotheses are proposed:

H1: eHL and MHL have a significant negative effect on adolescent SMA and DASS.

H2: MeHL mediates the effect of eHL and MHL on adolescent SMA and DASS.

H3: MHL significantly moderates the effect of eHL on MeHL and, through a dual moderation mediation pathway, influences SMA and DASS.

## Methods

### Study Design

This study used a cross-sectional design with a stratified cluster random sampling method to examine the relationships between adolescents' multidimensional health literacy and their engagement with SMA, as well as symptoms of depression, anxiety, and stress. The study was reported in accordance with the STROBE (Strengthening the Reporting of Observational Studies in Epidemiology) statement [50].

### Setting

This cross-sectional study was conducted in 5 provinces of China (Beijing, Zhejiang, Shanxi, Henan, and Jiangsu), selected to represent a range of regional digitalization levels. Data collection was carried out in the computer labs or classrooms of the participating schools over a 3-month period, from February to April 2025.

### Participants

The sampling procedure was conducted in 3 stages: first, 5 provinces were selected as strata based on regional digitalization indices; second, 2 general high schools were randomly selected within each province; and finally, 2 intact classes were randomly chosen from each school, resulting in a total of 20 classes. The target population comprised adolescents attending school, with exclusion criteria including severe physical or mental disabilities, inability to independently complete the questionnaire, or ongoing related treatment. A total of 855 adolescents completed valid questionnaires (response rate 85.5%), with 449 (52.5%) males, 406 (47.5%) females, and a mean age of 16.38 (SD 0.86) years.

### Variables

This study examined the following variable classes, all derived from self-report questionnaires:

- Outcome variables: SMA and a composite score of DASS served as the primary outcomes.
- Exposure variables: eHL and MHL were the core independent variables.
- Mediating variable: MeHL was tested as the mediator in the hypothesized pathways.
- Effect modifier: MHL was additionally examined as a moderator of the relationship between eHL and MeHL.
- Covariates: Participant age and gender were included as control variables in all multivariable analyses.

### Measurement

#### eHealth Literacy Scale

The eHealth Literacy Scale (eHEALS) developed by Norman and Skinner [22] was used. This scale contains 8 items and covers 3 dimensions: the ability to acquire, evaluate, and apply health information online. It assesses adolescents' knowledge, comfort, and self-perceived ability to acquire, understand, assess, and apply electronic health information. The scale uses a 5-point Likert scale, with responses ranging from 1 (“strongly disagree”) to 5 (“strongly agree”). The total score ranges from 8 to 40, with higher scores indicating higher levels of eHL. In this study, the scale demonstrated excellent internal consistency, with both a Cronbach α of 0.944 and a McDonald ω of 0.959 for the total score. The consistency estimates for the 3 dimensions were also high: application ability (α=0.907; ω=0.948), evaluation ability (α=0.883; ω=0.934), and decision-making ability (α=0.809; ω=0.922).

#### Mental Health Literacy Scale

MHL was assessed using a 12-item short form adapted for this study from the original scale by O'Connor and Casey [21]. To ensure brevity, we selected items that best represented the 4 theoretical dimensions: problem recognition, willingness to seek help, knowledge understanding, and attitudes toward professional help. The measure uses a 5-point Likert scale (“1=strongly disagree” to “5=strongly agree”), and a sum score is calculated (range 12-60), with higher scores reflecting higher MHL. The validity and reliability of this adapted form were supported empirically in our sample: its structural validity was upheld by confirmatory factor analysis (CFA). It demonstrated high internal consistency, with a Cronbach α of 0.946 and a McDonald ω of 0.964 for the total score. The coefficients for the 4 dimensions were problem recognition (α=0.885; ω=0.942), help-seeking (α=0.838; ω=0.919), knowledge understanding (α=0.867; ω=0.941), and attitude (α=0.901; ω=0.949).

#### Mental eHealth Literacy Scale

The Mental eHealth Literacy Scale (MeHLS) developed by Richard [23] was used. This scale consists of 23 items, divided into 3 dimensions: cognitive (8 items), knowledge (7 items), and skills (8 items), to assess an individual's knowledge, beliefs, and online information processing abilities in managing mental health. The scale uses a 5-point Likert scale (“1=never” to “5=always”), with a total score range of 23 to 115. Higher scores indicate higher levels of digital MHL. In this study, the internal consistency was excellent, with a Cronbach α of 0.953 and a McDonald ω of 0.960 for the total scale. The values for the 3 dimensions were cognitive (α=0.933; ω=0.949), knowledge (α=0.929; ω=0.943), and skills (α=0.937; ω=0.948).

#### Social Media Addiction Scale

The Bergen Social Media Addiction Scale (BSMAS), developed by Banyai [37], was used for assessment. This scale consists of 6 items reflecting 6 typical addiction characteristics: salience, emotional regulation, tolerance, withdrawal symptoms, conflict, and relapse [51,52]. In line with its widespread application and robust psychometric validation across different cultures and adolescent samples, the BSMAS is consistently treated as a unidimensional scale, and the sum of all items is used to indicate the severity of SMA tendencies [53-55]. It is used to identify adolescents' problematic social media use behaviors. The scale uses a 5-point Likert scale (“1=never” to “5=always”), with a total score range of 6 to 30. Higher scores indicate stronger tendencies toward SMA. In this study, the internal consistency for the scale was good, as indicated by both Cronbach α (0.894) and McDonald ω (0.928).

#### Depression-Anxiety-Stress Scale

The Depression Anxiety Stress Scale-21 (DASS-21), developed by Lovibond in 1995 and later revised and optimized by Antony, was used. This scale contains 21 items divided into 3 subscales—depression, anxiety, and stress—to assess the severity of emotional symptoms over the past week. The scale uses a 4-point Likert scale (“0=never” to “3=always”), with higher scores on each subscale indicating more severe symptoms. For the purpose of this study, a composite *psychological distress* total score was computed by summing the scores from the three DASS-21 subscales (depression, anxiety, and stress). For the primary analyses (including correlation and mediation analysis), a composite “psychological distress” total score was computed by summing the scores of the 3 subscales. The scale demonstrated excellent reliability in our sample. The internal consistency estimates for the subscales were as follows: depression (Cronbach α=0.909; McDonald ω=0.946), anxiety (α=0.898; ω=0.939), and stress (α=0.914; ω=0.947). The psychological distress total score also showed excellent internal consistency (α=0.959; ω=0.963).

The scales used in this study were all based on mature tools developed both domestically and internationally, or were revised and localized based on previous research. They are grounded in clear theoretical frameworks, scientifically structured designs, and supported by solid practical applications. The full survey questionnaire is available in Multimedia Appendix 1. To assess the construct validity of the scales, CFA was conducted to evaluate the goodness-of-fit of each measurement model.

The results showed that all models met the evaluation criteria: the *χ*^2^/*df* ratio was within a reasonable range, and the robust comparative fit indices (comparative fit index, Tucker-Lewis index, and incremental fit index) consistently exceeded the recommended threshold of 0.90, with most surpassing 0.95. While the goodness-of-fit index and adjusted goodness-of-fit index for the more complex DASS-21 model were slightly below 0.90—a common occurrence for multidimensional scales—the collective evidence strongly supports model fit. The root-mean-square error of approximation was less than 0.05, and the factor loadings were all at moderate to high levels, indicating that the scales have good construct validity. In conclusion, the measurement tools used in this study meet psychometric standards in terms of reliability and validity, ensuring high measurement quality and providing a solid quantitative foundation for subsequent empirical analyses.

### Bias

To address potential sources of bias, several strategies were implemented, with a specific focus on common method bias given the exclusive reliance on self-reported data. Procedural remedies during the research design and data collection phases included anonymous completion, randomization of questionnaire items, and the use of scales with varied response formats to mitigate the influence of potential source bias at the outset. Statistically, Harman single-factor test was planned and used to empirically evaluate the presence of common method bias [56].

### Study Size

The study size was determined by both feasibility and statistical considerations. Given the constraints of the stratified cluster sampling design, we aimed to recruit a feasible sample from the accessible schools in 5 provinces. The final analytic sample consisted of 855 adolescents. To confirm the adequacy of this achieved sample, a post hoc power analysis was conducted using G*Power 3.1. For a linear multiple regression with 8 predictors, an alpha of 0.05, and a medium effect size (*f*^2^=0.15), our sample provided over 95% power, substantially exceeding the minimum requirement of 160 participants, ensuring sufficient power to detect medium and small effect sizes.

### Statistical Methods

Data analysis was performed using IBM SPSS 20.0 for tests of common method bias, descriptive statistics, and correlation analysis; IBM Amos 24.0 for CFA and structural equation modeling (SEM); and SPSS PROCESS v4.1 macro for testing mediation and moderation effects. To account for the nonindependence of observations arising from the clustered sampling design, all primary analyses (including SEM and moderated mediation) were conducted using cluster-robust SEs with the classroom as the cluster unit. This method provides corrected SEs and CIs, mitigating the risk of type I error.

A total of 1000 questionnaires were distributed, with 893 returned, resulting in a response rate of 89.3%. The 107 nonreturned questionnaires were primarily attributed to student absences or leave during the survey period. The 893 returned questionnaires were screened for validity and missing data through a 2-stage process. First, to handle missing data, incomplete questionnaires (n=28) with any missing items were excluded. The extent of missing data was minimal, with item-level missingness ranging from 0.2% to 0.5% across all study variables. Little's test for missing completely at random (MCAR) was nonsignificant (*χ*^2^_85_=98.76, *P*=.15). Given that the data met the MCAR assumption and the fraction of incomplete cases was very small, the use of complete-case analysis was considered methodologically appropriate for this step [57].

Subsequently, a second stage of screening for data quality was performed on the remaining complete cases, leading to the exclusion of an additional 10 questionnaires due to abnormal completion times (n=7) or patterned responses (n=3) [58]. Following this rigorous process, 855 valid and complete questionnaires were retained for all subsequent analyses, representing 95.7% of returned surveys.

### Ethical Considerations

This study was conducted in strict accordance with ethical principles for research involving human participants. The study protocol was reviewed and approved by the Academic Ethics Committee of Capital University of Physical Education and Sports (approval no. 2025A0105). Prior to data collection, written informed consent was obtained from all participating adolescents and their parents or legal guardians. The consent process was conducted through the schools. Specifically, information sheets and consent forms detailing the study's purpose, procedures, potential risks and benefits, and the right to withdraw at any time without penalty were distributed to parents via students. After parental consent was secured, the same documents were presented to the adolescents in an age-appropriate manner to seek their assent.

All data were collected and processed anonymously, with no personally identifiable information stored, to ensure participant privacy and confidentiality. Data were used solely for the purposes of this academic research. Participants did not receive any monetary or material compensation for their involvement in the study. Finally, this manuscript does not contain any images that could lead to the identification of an individual participant.

## Results

### Common Method Bias Test

The results of the Harman single-factor test were examined to assess the threat of common method bias. The unrotated exploratory factor analysis revealed 8 factors with eigenvalues greater than 1. The first factor accounted for 31.07% of the total variance, which is significantly below the critical threshold of 40%. This indicates that no single factor explained the majority of the variance, suggesting that common method bias is not a severe issue in this dataset and does not pose a substantial threat to the interpretation of the study's findings. Furthermore, the CFA demonstrated good psychometric properties for all measurement scales, confirming their construct validity (Table 1).

**Table 1:** Confirmatory factor analysis results for all measurement scales in the adolescent sample (N=855).

Measurement scales	*χ*^2^/*df*	GFI^a^	AGFI^b^	RMSEA^c^	SRMR^d^	NFI^e^	IFI^f^	CFI^g^	TLI^h^	Factor loadings range
eHEALS^i^	1.768	0.996	0.981	0.030	0.032	0.998	0.999	0.999	0.996	0.753-0.928
MHLS-SF^j^	2.711	0.982	0.958	0.045	0.042	0.989	0.993	0.993	0.986	0.753-0.887
MeHLS^k^	2.943	0.942	0.922	0.048	0.045	0.965	0.977	0.977	0.971	0.711-0.883
BSMAS^l^	2.972	0.998	0.976	0.048	0.047	0.998	0.999	0.999	0.999	0.536-0.894
DASS^m^	2.774	0.809	0.792	0.046	0.052	0.883	0.922	0.922	0.917	0.628-0.840

^a^GFI: goodness-of-fit index.

^b^AGFI: adjusted goodness-of-fit index.

^c^RMSEA: root-mean-square error of approximation.

^d^SRMR: standardized root-mean-square residual.

^e^NFI: normed fit index.

^f^IFI: incremental fit index.

^g^CFI: comparative fit index.

^h^TLI: Tucker-Lewis index.

^i^eHEALS: eHealth Literacy Scale.

^j^MHLS-SF: Mental Health Literacy Scale – Short Form.

^k^MeHLS: Mental eHealth Literacy Scale.

^l^BSMAS: Bergen Social Media Addiction Scale.

^m^DASS: Depression-Anxiety-Stress Scale.

### Intraclass Correlation and Clustering Effects

Prior to testing our hypotheses, we quantified the clustering effect by calculating the intraclass correlation coefficient (ICC) for our primary outcome variables. The analysis revealed that 8.5% of the total variance in SMA (ICC=0.085, 95% CI 0.043-0.150) and 6.2% of the variance in DASS (ICC=0.062, 95% CI 0.028-0.125) resided between classrooms, justifying the need to account for the clustered data structure. All subsequent analyses used cluster-robust SEs to ensure valid statistical inference.

### Descriptive Statistics and Pearson Correlation Analysis

Descriptive statistics and correlation coefficients for each variable are presented in Table 2. The results revealed significant positive correlations between eHL, MHL, and MeHL. Specifically, eHL showed a moderate to strong positive correlation with both MHL and MeHL. MHL and MeHL also demonstrated a significant positive correlation. Furthermore, all 3 health literacy variables were significantly negatively correlated with SMA and DASS, with these relationships being statistically significant. This suggests that individuals with higher health literacy levels tend to experience lower levels of SMA and psychological distress related to DASS. Gender was significantly correlated with some variables, while age showed relatively weaker correlations. Therefore, gender and age were included as control variables in subsequent analyses to enhance the accuracy and robustness of the results.

**Table 2:** Descriptive statistics and correlations among study variables in Chinese high school students (N=855)^a^.

Variable	Mean (SD)	Gender	Age	eHL^b^	MHL^c^	MeHL^d^	SMA^e^	DASS^f^
Gender	1.47 (0.500)	1						
Age	16.38 (0.865)	–0.062 (–0.130 to 0.007)	1					
eHL	26.63 (7.928)	0.029 (–0.039 to 0.096)	0.042 (–0.023 to 0.109)	1				
MHL	46.74 (10.184)	0.089^g^ (0.024 to 0.155)	–0.020 (–0.085 to 0.045)	0.547^g^ (0.495 to 0.596)	1			
MeHL	70.25 (17.457)	0.030 (–0.035 to 0.096)	0.081^h^ (0.010 to 0.153)	0.695^g^ (0.659 to 0.729)	0.579^g^ (0.528 to 0.623)	1		
SMA	15.72 (6.026)	–0.015 (–0.082 to 0.052)	–0.022 (–0.045 to 0.087)	–0.369^g^ (–0.432 to –0.303)	–0.400^g^ (–0.462 to –0.335)	–0.370^g^ (–0.430 to –0.309)	1	
DASS	39.59 (14.649)	–0.031 (–0.096 to 0.036)	–0.009 (–0.073 to 0.056)	–0.433^g^ (–0.491 to –0.373)	–0.658^g^ (–0.706 to –0.604)	–0.444^g^ (–0.502 to –0.381)	0.536^g^ (0.479 to 0.591)	1

^a^Gender was dummy-coded, with 1 representing male and 2 representing female. The 95% CIs for the Pearson correlation coefficients, shown in parentheses, were derived from 5000 bootstrap samples. The correlation coefficients in the table are based on 2-tailed tests; the Pearson correlation coefficients are presented below the diagonal.

^b^eHL: eHealth literacy.

^c^MHL: mental health literacy.

^d^MeHL: mental eHealth literacy.

^e^SMA: social media addiction.

^f^DASS: depression-anxiety-stress.

^g^*P*<.01.

^h^*P*<.05.

### Mediation Effect Test

To further explore the mechanisms of the associations between eHL, MHL, and SMA among high school students and to examine the potential mediating effect of MeHL, the study constructed an SEM path analysis. The chi-square statistic was significant (*χ*^2^_2276_=6102.464, *P*<.001); thus, the normed chi-square (*χ*^2^/*df*) was consulted [59]. The fit indices of the revised model indicated an acceptable to good fit with the data: *χ*^2^/*df* (2.681), root-mean-square error of approximation (0.044), standardized root-mean-square residual (0.048), normed fit index (0.895), incremental fit index (0.931), comparative fit index (0.931), Tucker-Lewis index (0.927), and coefficient of determination (0.85) [60]. Although the goodness-of-fit index (0.814) and the adjusted goodness-of-fit index (0.797) were slightly below the commonly used threshold (0.90), the model still demonstrated good fit based on the other major fit indices, supporting further analysis of the relationships between the variables [61,62].

**Figure 2:**
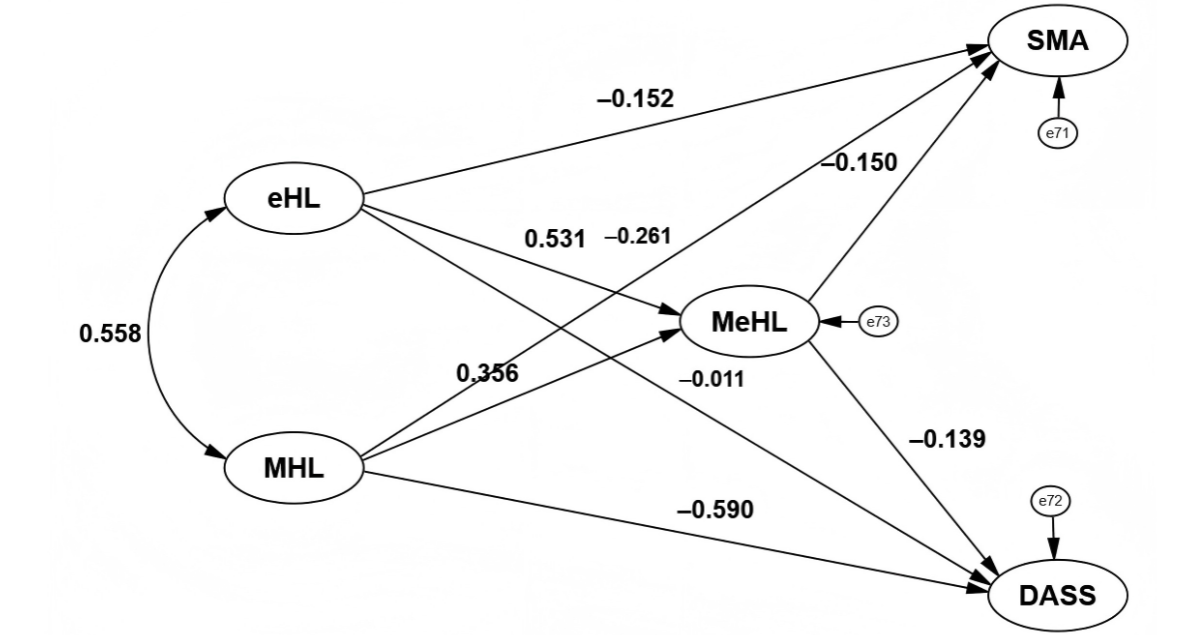
Structural equation modeling path diagram testing the mediating role of mental eHealth literacy. DASS: depression-anxiety-stress; eHL: eHealth literacy; MeHL: mental eHealth literacy; MHL: mental health literacy; SMA: social media addiction.

As shown in Figure 2 and Table 3, both eHL and MHL were significantly and positively associated with MeHL (β=0.531, 95% CI 0.468-0.594, *P*<.001; β=0.356, 95% CI 0.297-0.415, *P*<.001). Both were significantly negatively associated with SMA (β=–0.152, 95% CI –0.242 to –0.062, *P*=.01; β=–0.261, 95% CI –0.345 to –0.177, *P*<.001). However, eHL did not have a significant effect on DASS (β=–0.011, 95% CI –0.060 to 0.038; *P*=.81), while MHL was a significant negative predictor of DASS (β=–0.590, 95% CI –0.647 to –0.533; *P*<.001). Additionally, MeHL was also a significant negative predictor of SMA and psychological distress (β=–0.150, 95% CI –0.277 to –0.023, *P*=.02; β=–0.139, 95% CI –0.207 to –0.071, *P*=.006).

**Table 3:** Standardized direct path coefficients from the structural equation model^a^.

Path	Estimate, β (95% CI)	SE	CR^b^	*P* value
eHL^c^ → MeHL^d^	0.531 (0.468 to 0.594)	0.032	12.188	<.001
MHL^e^ → MeHL	0.356 (0.297 to 0.415)	0.030	9.433	<.001
eHL → SMA^f^	–0.152 (–0.242 to –0.062)	0.046	–2.543	.01
MHL → SMA	–0.261 (–0.345 to –0.177)	0.043	–5.093	<.001
eHL → DASS^g^	–0.011 (–0.060 to 0.038)	0.025	–0.240	.81
MHL → DASS	–0.590 (–0.647 to –0.533)	0.029	–11.241	<.001
MeHL → SMA	–0.150 (–0.277 to –0.023)	0.065	–2.431	.02
MeHL → DASS	–0.139 (–0.207 to –0.071)	0.035	–2.743	.006

^a^All parameters estimated with cluster-robust SEs (clustered by classroom).

^b^CR: critical ratio.

^c^eHL: eHealth literacy.

^d^MeHL: mental eHealth literacy.

^e^MHL: mental health literacy.

^f^SMA: social media addiction.

^g^DASS: depression-anxiety-stress.

To further examine the mediation effects, we calculated the fully standardized indirect effects and their 95% bias-corrected CIs using bootstrap sampling with 5000 resamples and cluster-robust SEs (Table 4). The results indicated that both eHL and MHL exerted significant negative indirect effects on SMA through MeHL, as the 95% CIs for these paths did not contain zero. Specifically, the indirect effect of eHL was β=–0.062, 95% CI –0.125 to –0.010, and that of MHL was β=–0.045, 95% CI –0.090 to –0.008. In practical terms, this suggests that for every one standard deviation increase in eHL, SMA is expected to decrease by 0.062 standard deviations via the mediator. Similarly, the indirect effects on DASS were also significant (eHL: β=–0.037, 95% CI –0.072 to –0.009; MHL: β=–0.027, 95% CI –0.053 to –0.007). The proportion of the total effect mediated (relative effect size) for each path is also presented in Table 4.

**Table 4:** Bootstrap analysis of indirect effects through mental eHealth literacy^a^.

Path and effect type			Estimate, β (95% CI)		*P* value		Proportion mediated
**eHL^b^→ MeHL^c^→ SMA^d^**							34.4%
	Mediation	–0.062 (–0.125 to –0.010)		.01			
	Total	–0.179 (–0.258 to –0.105)		.001			
**MHL^e^→ MeHL → SMA**							17%
	Mediation	–0.045 (–0.090 to –0.008)		.01			
	Total	–0.264 (–0.350 to –0.188)		.001			
**eHL → MeHL → DASS^f^**							86.6%
	Mediation	–0.037 (–0.072 to –0.009)		.008			
	Total	–0.043 (–0.087 to –0.004)		.03			
**MHL → MeHL → DASS**							77%
	Mediation	–0.027 (–0.053 to –0.007)		.007			
	Total	–0.353 (–0.422 to –0.290)		.001			

^a^Bootstrap CIs and *P* values are based on cluster-robust SEs (clustered by classroom). Estimates are fully standardized indirect effects (β).

^b^eHL: eHealth literacy.

^c^MeHL: mental eHealth literacy.

^d^SMA: social media addiction.

^e^MHL: mental health literacy.

^f^DASS: depression-anxiety-stress.

In summary, the research results are consistent with a model in which MeHL plays a potential mediating role in the associations linking eHL and MHL to adolescent SMA and DASS. Specifically, higher levels of eHL and MHL were associated with higher MeHL, which in turn was linked to lower levels of SMA and psychological distress related to DASS. The study suggests a potential synergistic pathway of the “eHealth–mental health” dual-track mechanism that may be relevant for interventions targeting SMA and psychological distress, offering significant theoretical value and practical implications.

### Moderation Effect Analysis

To explore the interaction between eHL and MHL in predicting MeHL, the study used a stepwise regression analysis with cluster-robust SEs to construct a 3-stage progressive model (Table 5). Model 1 serves as the baseline model, controlling for gender and age. The results show that demographic variables explain very little of the variance in MeHL (*R*^2^=0.008), though the overall model was not statistically significant (*F*=3.344, *P*<.05). Model 2 introduces eHL and MHL as predictors. Both have a significant positive effect on MeHL (eHL: β=0.535, 95% CI 0.472-0.598, *P*<.01; MHL: β=0.288, 95% CI 0.229-0.347, *P*<.01). The model's explanatory power significantly increases (*R*^2^=0.544), with a Δ*R*^2^=0.536 compared to model 1, *F*=253.232, *P*<.001, indicating that these 2 forms of health literacy are important predictors of MeHL. Model 3 adds the interaction term of eHL and MHL (centered). The results show that the interaction term's regression coefficient is significant (β=0.067, 95% CI 0.010-0.124; *P*<.05), and the model's explanatory power slightly increases (*R*^2^=0.547, Δ*R*^2^=0.003, *F*=205.285, *P*<.001), indicating a synergistic effect between the two, enhancing their ability to predict MeHL.

**Table 5:** Hierarchical regression analysis testing the interaction between eHealth literacy and mental health literacy^a^.

Variable	MeHL^b^		
	Model 1	Model 2	Model 3
Gender	0.036 (–0.032 to 0.104)	–0.007 (–0.055 to 0.041)	–0.004 (–0.052 to 0.044)
Age	0.083 (0.019 to 0.147)	0.064 (0.016 to 0.112)	0.063 (0.015 to 0.111)
eHL^c^		0.535^d^ (0.472 to 0.598)	0.524^d^ (0.461 to 0.587)
MHL^e^		0.288^d^ (0.229 to 0.347)	0.322^d^ (0.263 to 0.381)
eHL × MHL			0.067^f^ (0.010 to 0.124)
*R* ^2^	0.008	0.544	0.547
Δ*R*^2^	—	0.536	0.003
F	3.344^f^	253.232^g^	205.285^g^

^a^All models estimated with cluster-robust SEs (clustered by classroom). All reported coefficients are standardized regression coefficients (β) with 95% CIs in parentheses.

^b^MeHL: mental eHealth literacy.

^c^eHL: eHealth literacy.

^d^*P*<.01.

^e^MHL: mental health literacy.

^f^*P*<.05.

^g^*P*<.001.

**Figure 3:**
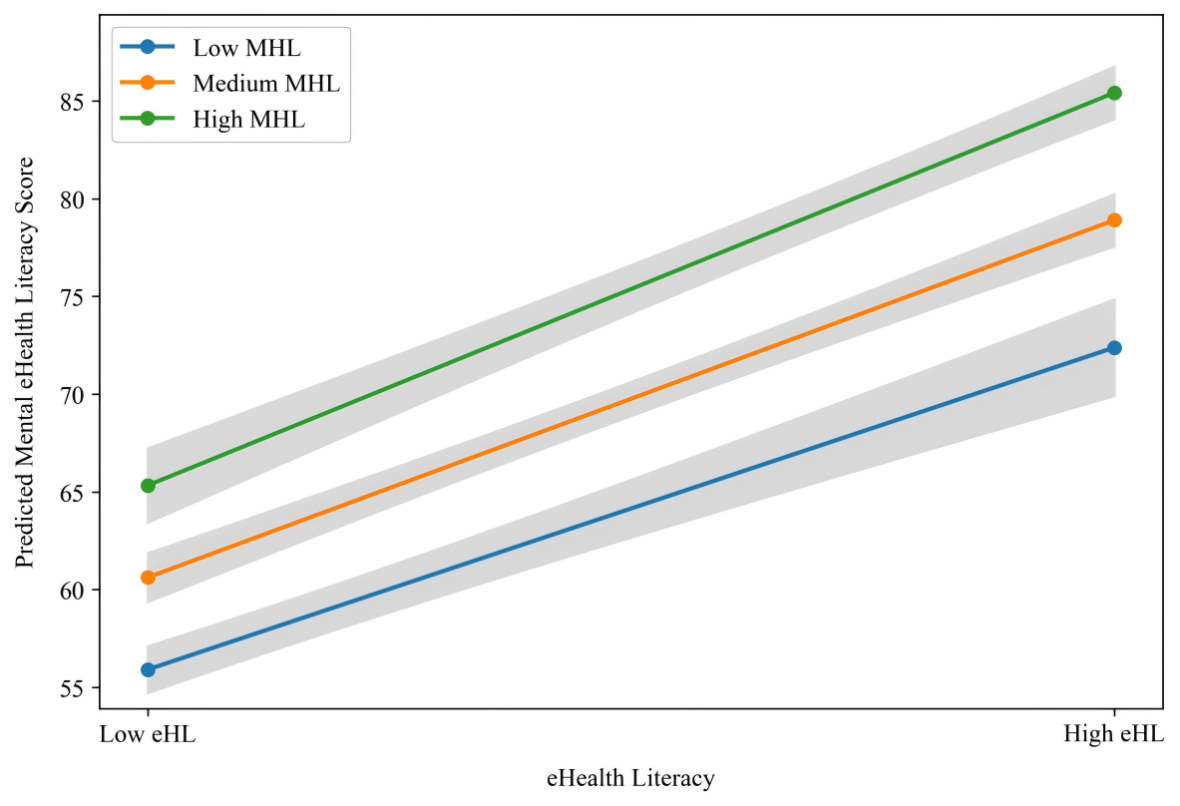
The moderating effect of mental health literacy on the relationship between eHealth literacy and mental eHealth literacy. eHL: eHealth literacy; MHL: mental health literacy.

To further clarify the nature of the interaction, a simple slope analysis was conducted to probe the association between eHL and MeHL at low (mean – 1 SD), medium (mean), and high (mean + 1 SD) levels of MHL (Figure 3). The analysis revealed a significant positive association between eHL and MeHL at low (*b*=1.040, 95% CI 0.881-1.199), medium (*b*=1.154, 95% CI 1.033-1.275), and high (*b*=1.268, 95% CI 1.131-1.405) levels of MHL. As visually represented in Figure 3, which plots the predicted MeHL scores and their CIs, these results demonstrate a clear synergistic pattern: the positive relationship between eHL and MeHL grows progressively stronger as the level of MHL increases.

### Moderated Mediation Effect Test

The study used the PROCESS (model 7) model proposed by Hayes in 2022 to test the mediation mechanism of MeHL in the relationship between eHL and SMA, as well as the moderating effect of MHL on this mediation path. In the analysis, all continuous predictor variables (ie, eHL and MHL) were mean-centered to reduce the impact of multicollinearity on the estimation of interaction terms. The bootstrap method was used for 5000 resamples to generate 95% bias-corrected CIs to assess the significance and stability of the moderated mediation effect. The Johnson-Neyman technique was used to precisely characterize the moderation pattern across the full range of MHL levels.

The moderated mediation analysis results (Table 6), estimated with cluster-robust SEs, demonstrate a consistent strengthening of indirect effects with increasing MHL levels. For SMA, the standardized indirect effect intensified from β=–0.084 at the 16th percentile to β=–0.099 at the 84th percentile of MHL, with a statistically significant pairwise contrast between high and low levels (Δ=–0.016, 95% CI –0.038 to –0.006). A more pronounced pattern was observed for DASS, where the indirect effect increased from β=–0.252 to β=–0.299 across the same percentile range, supported by a significant pairwise contrast (Δ=–0.047, 95% CI –0.102 to –0.008). The Johnson-Neyman analysis further confirmed that these mediation pathways remain statistically significant throughout the complete observed range of MHL, with no significant transition points identified. These findings indicate that higher levels of MHL significantly enhance the protective indirect effect of eHL on both behavioral and psychological outcomes through MeHL.

**Table 6:** Conditional indirect effects at different levels of mental health literacy (moderated mediation results)^a^.

Outcome variable, contrast, and MHL^b^ percentile				Effect size, β (95% CI)
**eHL^c^→ MeHL^d^→ SMA^e^**				
	**Conditional indirect effect**			
		Low (16th)	–0.084^f^ (–0.142 to –0.031)	
		Medium (50th)	–0.091^f^ (–0.152 to –0.035)	
		High (84th)	–0.099^f^ (–0.166 to –0.037)	
	Pairwise contrast (high vs low), Δ (95% CI)		–0.016^g^ (–0.038 to 0.006)	
**eHL → MeHL → DASS^h^**				
	**Conditional indirect effect**			
		Low (16th)	–0.252^f^ (–0.358 to –0.151)	
		Medium (50th)	–0.273^f^ (–0.382 to –0.169)	
		High (84th)	–0.299^f^ (–0.418 to –0.185)	
	Pairwise contrast (high vs low), Δ (95% CI)		–0.016^g^ (–0.038 to 0.006)	

^a^All values are standardized coefficients (β). CIs and significance tests are based on cluster-robust SEs (clustered by classroom).

^b^MHL: mental health literacy.

^c^eHL: eHealth literacy.

^d^MeHL: mental eHealth literacy.

^e^SMA: social media addiction.

^f^*P*<.01.

^g^*P*<.05.

^h^DASS: depression-anxiety-stress.

## Discussion

### Principal Findings

This study proposed and tested a multidimensional health literacy model to elucidate the synergistic pathways through which eHL and MHL influence adolescent SMA and DASS, with MeHL as a mediator and MHL as a moderator. Our findings, supported by robust bootstrap CIs, provide precise estimates of these complex relationships. In support of our hypotheses, the findings revealed that (1) H1 was partially supported, as both eHL and MHL were significant negative predictors of SMA, with CIs indicating stable negative associations, but only MHL was a significant negative predictor of DASS, while the nonsignificant path from eHL to DASS suggests a negligible direct effect; (2) H2 was fully supported, with MeHL serving as a significant mediator in the relationships from both eHL and MHL to SMA and DASS, with all indirect effect CIs excluding zero; and (3) H3 was also supported, as MHL positively moderated the effect of eHL on MeHL, indicating a statistically significant though modest synergistic interaction that strengthened the proposed moderated mediation pathways to the outcomes.

Delving into the distinct associations posited in H1, our findings clarify the unique roles of different health literacies. These differential pathways align with yet extend previous research that has often examined these literacies in isolation. MHL demonstrated a robust negative association with DASS, suggesting it serves as a primary psychological resource for emotional regulation [63], effectively breaking the “negative emotions–compensatory use” cycle [64,65]. This is consistent with population-based surveys that have identified MHL as a key correlate of psychological well-being in adolescents [31]. Conversely, eHL showed a more targeted association with reducing behavioral risks (SMA), equipping adolescents to navigate algorithmic “information cocoons” and use practical strategies like time management [66,67]. This finding resonates with large-scale studies linking digital competency to lower risks of problematic online behaviors, highlighting the role of eHL in mitigating specific behavioral risks rather than broad emotional distress [37]. Together, these dual pathways illustrate how distinct health literacies serve as protective resources. This mechanism aligns with the COR theory, as both literacies help adolescents conserve mental resources and mitigate digital threats [68]. Therefore, enhancing multidimensional health literacy constructs a dual barrier of “cognitive defense–behavioral regulation,” providing concrete intervention entry points.

The mediating role of MeHL (H2) represents a crucial synthesis of digital capability and psychological cognition. Our bootstrap analysis provides precise estimates of this integration, showing significant indirect effects from both eHL and MHL through MeHL. This result is consistent with the “triadic reciprocal model” in SCT, where personal factors (MHL), behaviors, and the digital environment interact continuously [69]. eHL provides the tool for information access, while MHL provides the cognitive framework for interpretation; their confluence fosters MeHL. This integration is critical, as individuals must not only find digital health information but also critically evaluate and apply it. The ability to identify and resist health misinformation is a key component of advanced health literacy, which our MeHL construct appears to capture [70]. Furthermore, general health literacy has been associated with more sophisticated information processing strategies, which may underpin the effective integration of digital and mental health knowledge reflected in MeHL [71]. Notably, the stronger mediation effect for eHL suggests the digital capability pathway is particularly dependent on this integrative process. MeHL thus plays a key role in the “knowledge internalization–behavioral transformation” process, offering a layered intervention path.

Most importantly, the supported moderated mediation hypothesis (H3) reveals a dynamic synergy at the heart of our model. The positive moderation effect indicates that higher MHL levels strengthen the effect of eHL on MeHL. This finding offers strong support for COR theory's principle of resource caravans [72], wherein existing resources (MHL) facilitate the acquisition and transformation of new resources (eHL). This boosting effect makes sense when we consider teenage brain development. Teenagers' brains are especially tuned to seek rewards and are highly motivated to learn new things that help them adapt [73]. In this context, MHL may provide the necessary motivational and cognitive framework—the “why”—making the technical skills of eHL—the “how”—more valuable and worth applying to mental health contexts, thereby enhancing MeHL. This positions MHL as a “cognitive catalyst” that unlocks digital skills' potential. Individuals with high MHL show greater cognitive processing depth when dealing with digital health information, while those with low MHL struggle to achieve behavioral transformation despite technical skills [73].

In conclusion, this study challenges the siloed perspective of previous research by proposing and validating a comprehensive model that elucidates the dynamic interplay between different health literacies. Unlike earlier work that primarily examined eHL and MHL in isolation, our findings demonstrate that their synergistic association—where MHL catalytically enhances the utility of eHL—is crucial for understanding adolescent outcomes in the digital age. This integrated perspective is supported by research indicating that combined literacy interventions can be more effective, and our model provides a specific mechanistic account of this synergy [46]. This validated dual-path moderated mediation model provides a significant theoretical advancement by moving beyond direct effects to reveal a more complex relational network. Methodologically, our robust approach accounting for clustered data strengthens the ecological validity of these observed associations. These insights carry direct practical implications, providing an evidence-based blueprint for integrated health promotion that concurrently targets psychological cognition and digital competencies. Fostering this synergy represents a promising and efficient strategy for safeguarding adolescent well-being and building a healthier digital future.

### Limitations

Notwithstanding its contributions, this study has several limitations that should be considered. The cross-sectional design precludes causal inference, leaving open the possibility of reverse causality, such as the potential for psychological distress to diminish health literacy acquisition. While common method bias was statistically assessed and fell below critical thresholds, the use of self-reported data may still introduce social desirability bias and inflate relationship estimates. Furthermore, the generalizability of the findings may be constrained by the regional sampling frame within China, and the omission of potential confounders such as socioeconomic status, academic pressure, and precise social media usage metrics means that the reported associations, though robust, may partially reflect the influence of these unmeasured variables. Future longitudinal studies incorporating objective behavioral data and a broader set of covariates in diverse cultural contexts are warranted to confirm the causal and generalizable nature of these synergistic pathways.

### Conclusions

This cross-sectional study validates a new model that overcomes the limitations of previous research, which typically examined health literacy dimensions in isolation. It reveals the key role of eHL and MHL in reducing SMA and alleviating DASS among adolescents. The study shows that while both eHL and MHL contribute to reducing SMA, only MHL directly alleviates DASS. Additionally, MeHL plays a critical mediating role in this process. The innovation of this study lies in demonstrating that MHL is not merely an independent protective factor but an important moderator that enhances the effect of eHL in promoting MeHL. MHL provides the cognitive foundation for the effective application of digital skills, enabling adolescents to better use digital tools to manage their mental health, thereby maximizing the protective potential of digital skills. Therefore, public health strategies should focus on integrated interventions that simultaneously enhance both psychological resilience and digital competence, which is crucial for safeguarding adolescent well-being.

## Appendix

Multimedia Appendix 1 Survey questionnaire.

Multimedia Appendix 2 STROBE checklist.

## Abbreviations

BSMAS: Bergen Social Media Addiction Scale
CFA: confirmatory factor analysis
COR: conservation of resources
DASS: depression-anxiety-stress
DASS-21: Depression Anxiety Stress Scale-21
eHEALS: eHealth Literacy Scale
eHL: eHealth literacy
HBM: health belief model
ICC: intraclass correlation coefficient
MCAR: missing completely at random
MeHL: mental eHealth literacy
MeHLS: Mental eHealth Literacy Scale
MHL: mental health literacy
SCT: social cognitive theory
SEM: structural equation modeling
SMA: social media addiction
STROBE: Strengthening the Reporting of Observational Studies in Epidemiology

## Data Availability

The deidentified datasets generated and analyzed during this study are not publicly available due to restrictions in the ethical approval aimed at protecting adolescent participant confidentiality. However, they are available from the corresponding author, Zuosong Chen, upon reasonable request. Requestors will be required to complete a data use agreement, committing to use the data solely for noncommercial academic research purposes. The analysis code (IAMOS syntax and PROCESS macro) is also available upon request.

## References

[1] (2019). Notice on issuing the action plan for Healthy China Initiative – children and adolescents' mental health (2019-2022). National Health Commission of the People's Republic of China.

[2] (2019). Healthy China Initiative (2019-2030). National Health Commission of the People's Republic of China.

[3] (2023). Notice on issuing the Special Action Plan for Comprehensively Strengthening and Improving Students' Mental Health Work in the New Era (2023-2025). Ministry of Education of the People's Republic of China.

[4] (2025). The Central Committee of the Communist Party of China and the State Council issued the "Outline of the Plan for Building a Strong Education Nation (2024-2035)". The State Council of the People's Republic of China.

[5] (2024). Caring for mental health and escorting sunshine growth—a summary of the education system's collaborative efforts to promote adolescent mental health work. Ministry of Education of the People's Republic of China.

[6] (2025). Improving adolescent mental health through social work professional services. Chinese Social Sciences Net.

[7] (2022). 2021 national research report on internet use by minors. China Youth Network.

[8] (2023). Beware of social media impacting adolescent mental health. Ministry of Education of the People's Republic of China.

[9] (2024). Improving people's health and building a healthy China—studying "Excerpts from Xi Jinping's Discourses on Healthy China". People's Daily Online.

[10] Sørensen K, Pelikan JM, Röthlin F, Ganahl K, Slonska Z, Doyle G, Fullam J, Kondilis B, Agrafiotis D, Uiters E, Falcon M, Mensing M, Tchamov K, van den Broucke S, Brand H, HLS-EU Consortium (2015). Health literacy in Europe: comparative results of the European Health Literacy Survey (HLS-EU). Eur J Public Health.

[11] (2025). Enhancing adolescent mental health literacy. Chinese Social Sciences Net.

[12] Friis K, Lasgaard M, Rowlands G, Osborne RH, Maindal HT (2016). Health literacy mediates the relationship between educational attainment and health behavior: a Danish population-based study. J Health Commun.

[13] Wang C, Kane RL, Xu D, Meng Q (2015). Health literacy as a moderator of health-related quality of life responses to chronic disease among Chinese rural women. BMC Womens Health.

[14] Hua Z, Yuqing S, Qianwen L, Hong C (2025). Factors influencing eHealth literacy worldwide: systematic review and meta-analysis. J Med Internet Res.

[15] Náfrádi L, Nakamoto K, Csabai M, Papp-Zipernovszky O, Schulz PJ (2018). An empirical test of the health empowerment model: does patient empowerment moderate the effect of health literacy on health status?. Patient Educ Couns.

[16] Meppelink CS, van Weert JCM, Haven CJ, Smit EG (2015). The effectiveness of health animations in audiences with different health literacy levels: an experimental study. J Med Internet Res.

[17] Nutbeam D (2008). The evolving concept of health literacy. Soc Sci Med.

[18] (2024). Health literacy. World Health Organization.

[19] Jorm AF, Korten AE, Jacomb PA, Christensen H, Rodgers B, Pollitt P (1997). "Mental health literacy": a survey of the public's ability to recognise mental disorders and their beliefs about the effectiveness of treatment. Med J Aust.

[20] Jorm AF (2012). Mental health literacy: empowering the community to take action for better mental health. Am Psychol.

[21] O'Connor M, Casey L (2015). The Mental Health Literacy Scale (MHLS): a new scale-based measure of mental health literacy. Psychiatry Res.

[22] Norman CD, Skinner HA (2006). eHEALS: the eHealth Literacy Scale. J Med Internet Res.

[23] Xu RH, Cao Y, Dong D, Wong EL, Chan SK (2024). Development and validation of a mental eHealth literacy scale. Comput Hum Behav Rep.

[24] Bröder J, Okan O, Bauer U, Bruland D, Schlupp S, Bollweg TM, Saboga-Nunes L, Bond E, Sørensen K, Bitzer E, Jordan S, Domanska O, Firnges C, Carvalho GS, Bittlingmayer UH, Levin-Zamir D, Pelikan J, Sahrai D, Lenz A, Wahl P, Thomas M, Kessl F, Pinheiro P (2017). Health literacy in childhood and youth: a systematic review of definitions and models. BMC Public Health.

[25] Xiangnan C (2024). Enhancing health literacy from multiple dimensions effectively shapes a healthy lifestyle. Chinese Social Sciences Net.

[26] Hua ZC, Sun J (2021). Effects of mind-body practice on anxiety, depression, and stress in college students: an experimental study. J Guangzhou Sport Univ.

[27] Yap MBH, Jorm AF (2011). The influence of stigma on first aid actions taken by young people for mental health problems in a close friend or family member: findings from an Australian national survey of youth. J Affect Disord.

[28] Rafal G, Gatto A, DeBate R (2018). Mental health literacy, stigma, and help-seeking behaviors among male college students. J Am Coll Health.

[29] Gorczynski P, Sims-schouten W, Hill DM, Wilson JC (2017). Examining mental health literacy, help seeking behaviours, and mental health outcomes in UK university students. J Ment Health Train Educ Pract.

[30] Brijnath B, Protheroe J, Mahtani KR, Antoniades J (2016). Do web-based mental health literacy interventions improve the mental health literacy of adult consumers? Results from a systematic review. J Med Internet Res.

[31] Lam LT (2014). Mental health literacy and mental health status in adolescents: a population-based survey. Child Adolesc Psychiatry Ment Health.

[32] Castarlenas E, Sánchez-Rodríguez E, Roy R, Tomé-Pires C, Solé E, Jensen MP, Miró J (2021). Electronic health literacy in individuals with chronic pain and its association with psychological function. Int J Environ Res Public Health.

[33] Yang BX, Xia L, Huang R, Chen P, Luo D, Liu Q, Kang LJ, Zhang Z, Liu Z, Yu S, Li X, Wang XQ (2021). Relationship between eHealth literacy and psychological status during COVID-19 pandemic: a survey of Chinese residents. J Nurs Manag.

[34] Xu RH, Shi L-S-B, Xia Y, Wang D (2022). Associations among eHealth literacy, social support, individual resilience, and emotional status in primary care providers during the outbreak of the SARS-CoV-2 Delta variant. Digit Health.

[35] Zhao Y, Zhao Y, Xie Z, Ziyadan P, Xiu Z, Qi M (2023). The relationship between eHealth literacy and mental health of adult workers: a cross-sectional study. J Psychosoc Nurs Ment Health Serv.

[36] Cao X, Gong M, Yu L, Dai B (2020). Exploring the mechanism of social media addiction: an empirical study from WeChat users. Internet Res.

[37] Bányai F, Zsila Á, Király O, Maraz A, Elekes Z, Griffiths MD, Andreassen CS, Demetrovics Z (2017). Problematic social media use: results from a large-scale nationally representative adolescent sample. PLoS One.

[38] Cheng C, Lau Y, Chan L, Luk JW (2021). Prevalence of social media addiction across 32 nations: meta-analysis with subgroup analysis of classification schemes and cultural values. Addict Behav.

[39] Tennant B, Stellefson M, Dodd V, Chaney B, Chaney D, Paige S, Alber J (2015). eHealth literacy and Web 2.0 health information seeking behaviors among baby boomers and older adults. J Med Internet Res.

[40] Hassen HM, Behera MR, Jena PK, Dewey RS, Disassa GA (2022). Effectiveness and implementation outcome measures of mental health curriculum intervention using social media to improve the mental health literacy of adolescents. J Multidiscip Healthc.

[41] Liu Y, Wu N, Yan J, Yu J, Liao L, Wang H (2023). The relationship between health literacy and internet addiction among middle school students in Chongqing, China: a cross-sectional survey study. PLoS One.

[42] Gioia F, Rega V, Boursier V (2021). Problematic internet use and emotional dysregulation among young people: a literature review. Clin Neuropsychiatry.

[43] Goldfus TB (2024). The impact of social media use on depression, anxiety, and well-being for teens/young people: using hypnosis to build a strong sense of self. Am J Clin Hypn.

[44] Lal S (2019). E-mental health: promising advancements in policy, research, and practice. Healthc Manage Forum.

[45] Cormier E, Park H, Schluck G (2022). College students' eMental health literacy and risk of diagnosis with mental health disorders. Healthcare (Basel).

[46] Yang J, Shen Q, Tong X, Mukhopadhaya P (2025). The impact of digital literacy in enhancing individuals' health in China. BMC Public Health.

[47] Tso WWY, Reichert F, Law N, Fu KW, de la Torre J, Rao N, Leung LK, Wang Y, Wong WHS, Ip P (2022). Digital competence as a protective factor against gaming addiction in children and adolescents: a cross-sectional study in Hong Kong. Lancet Reg Health West Pac.

[48] Zhang S, Wang W, Wu S, Ye H, Dong L, Wang J, Ning X, Cui H (2024). Analysis of the mediating effect between ehealth literacy and health self-management of undergraduate nursing students' mental health literacy. BMC Nurs.

[49] Hsu W, Chiang C, Yang S (2014). The effect of individual factors on health behaviors among college students: the mediating effects of eHealth literacy. J Med Internet Res.

[50] von Elm E, Altman DG, Egger M, Pocock SJ, Gøtzsche PC, Vandenbroucke JP, STROBE Initiative (2014). The Strengthening the Reporting of Observational Studies in Epidemiology (STROBE) statement: guidelines for reporting observational studies. Int J Surg.

[51] Shi YN, Zhang N, Yuan QJ (2020). Research on social media addiction abroad: measurement tools, theoretical models, and behavioral impacts. Modern Information.

[52] Luo T, Qin L, Cheng L, Wang S, Zhu Z, Xu J, Chen H, Liu Q, Hu M, Tong J, Hao W, Wei B, Liao Y (2021). Determination the cut-off point for the Bergen Social Media Addiction (BSMAS): diagnostic contribution of the six criteria of the components model of addiction for social media disorder. J Behav Addict.

[53] Bottaro R, Griffiths MD, Faraci P (2025). Meta-analysis of reliability and validity of the Bergen Social Media Addiction Scale (BSMAS). Int J Ment Health Addiction.

[54] Zarate D, Hobson BA, March E, Griffiths MD, Stavropoulos V (2023). Psychometric properties of the bergen social media addiction scale: an analysis using item response theory. Addict Behav Rep.

[55] Leung H, Pakpour AH, Strong C, Lin Y, Tsai M, Griffiths MD, Lin C, Chen I (2020). Measurement invariance across young adults from Hong Kong and Taiwan among three internet-related addiction scales: Bergen Social Media Addiction Scale (BSMAS), Smartphone Application-Based Addiction Scale (SABAS), and Internet Gaming Disorder Scale-Short Form (IGDS-SF9) (Study Part A). Addict Behav.

[56] Zhou H, Long LR (2004). Statistical remedies for common method biases. Adv Psychhol Sci.

[57] Little RJ, Carpenter JR, Lee KJ (2022). A comparison of three popular methods for handling missing data: complete-case analysis, inverse probability weighting, and multiple imputation. Sociol Methods Res.

[58] Zhong XY, Li MY, Li LY (2021). Control and identification of careless responding in questionnaire surveys. Adv Psychol Sci.

[59] Wen ZL, Hau KT, Marsh HW (2004). Structural equation model testing: cutoff criteria for goodness of fit indices and chi-square test. Acta Psychologica Sinica.

[60] Wen ZL, Zhang L, Hau KT, Liu HY (2004). Testing and application of the mediating effects. Acta Psychologica Sinica.

[61] Schermelleh-Engel K, Moosbrugger H, Müller H (2003). Evaluating the fit of structural equation models: tests of significance and descriptive goodness-of-fit measures. Methods Psychol Res.

[62] Hu LT, Bentler PM (1999). Cutoff criteria for fit indexes in covariance structure analysis: conventional criteria versus new alternatives. Struct Equ Model Multidiscip J.

[63] Chen Q, Zhao Z, Bao J, Lin J, Li W, Zang Y (2024). Digital empowerment in mental health: a meta-analysis of internet-based interventions for enhancing mental health literacy. Int J Clin Health Psychol.

[64] Xiao W, Cheng M (2023). The relationship between internet addiction and cyberbullying perpetration: a moderated mediation model of moral disengagement and internet literacy. Int J Ment Health Promot.

[65] Liu R, Xu SJ (2023). The impact of perceived stress on college students' e-health literacy: the mediating role of psychological resilience. J. Nanjing Med. Univ.

[66] Blackwell CK, Mansolf M, Rose T, Pila S, Cella D, Cohen A, Leve LD, McGrath M, Neiderhiser JM, Urquhart A, Ganiban JM (2025). Adolescent social media use and mental health in the environmental influences on child health outcomes study. J Adolesc Health.

[67] Wang X, Yue T, Mo PK (2022). The associations among cognitive social factors, eHealth literacy and health-promoting behaviors in Chinese adolescents. Health Promot Int.

[68] Cui X, Lei Y, Huo B, Lowry PB, Yang X (2025). Uncovering the effects of non-hedonic social media use on knowledge workers’ depression through the conservation of resources theory. Inf Manag.

[69] McAnally K, Hagger MS (2023). Health literacy, social cognition constructs, and health behaviors and outcomes: a meta-analysis. Health Psychol.

[70] Wang XY, Jin H (2025). Effective intervention strategies and their theoretical foundations for mitigating the continued influence effect of health misinformation. J Tianjin Univ.

[71] Peng RX, Shen F (2025). Why fall for misinformation? Role of information processing strategies, health consciousness, and overconfidence in health literacy. J Health Psychol.

[72] Wang H, Sun W, Zhou Y, Li T, Zhou P (2022). Teachers' assessment literacy improves teaching efficacy: a view from conservation of resources theory. Front Psychol.

[73] Kramer AW, Krabbendam L, Schaaf JV, Huizenga HM, Van Duijvenvoorde ACK (2025). Make it worth it: effort-reward modulations on reinforcement-learning and prediction-error signaling across adolescence. Dev Cogn Neurosci.

