# Supplementary material for: The Interactive Effects of eHealth Literacy and Mental Health Literacy on Social Media Addiction and Depression-Anxiety-Stress in Adolescents: Cross-Sectional Study
Source: J Med Internet Res. 2025 Nov 28;27:e81741. doi: 10.2196/81741 (PMC12701345; doi:10.2196/81741)
Supplement: Multimedia Appendix 1 [file jmir_v27i1e81741_app1.docx]

**Adolescent Mental Health Literacy Assessment Survey**

Dear Student,

Thank you for taking the time to participate in this research. This questionnaire aims to assess the mental health literacy of adolescents and provide a scientific basis for mental health education and interventions. Your participation is vital to our study, and we sincerely appreciate your support!

This survey is anonymous, and all data will be used solely for academic research purposes. Your personal information will be kept strictly confidential. Completing the questionnaire will take approximately 5-10 minutes. Please answer truthfully based on your actual experiences. There is no right or wrong answer. Your thoughtful responses will help us obtain accurate research results, which are of great significance to mental health research and practice.

Thank you again for your participation. We wish you success in your studies and happiness in life!

Thank you again for your participation. We wish you success in your studies and

**Section A: Basic Information**

Name： Gender：Male 🞎 Female🞎 Age： Grade：

Current location： Province / Autonomous Region / Municipality,

Prefecture-level City, District/County

**Section B: Mental Health Assessment**

**Mental Health Literacy Scale – Short Form (MHLS-SF)**

**（12 items）（Single-choice questions）**

|  | Strongly Disagree | Disagree | Neutral | Agree | Strongly Agree |
| --- | --- | --- | --- | --- | --- |
| 1. I can identify common mental health issues (e.g., depression, anxiety) |  |  |  |  |  |
| 1. I can differentiate between mental health problems and normal emotional fluctuations |  |  |  |  |  |
| 1. I understand the common manifestations of mental health problems |  |  |  |  |  |
| 1. If I encounter mental health issues, I am willing to seek help actively |  |  |  |  |  |
| 1. I know where to get mental health support |  |  |  |  |  |
| 1. If my friends or family have mental health issues, I will encourage them to seek professional help |  |  |  |  |  |
| 1. I believe mental health problems are treatable |  |  |  |  |  |
| 1. I know that mental health problems can be caused by various factors |  |  |  |  |  |
| 1. I think mental health is as important as physical health |  |  |  |  |  |
| 1. I trust mental health professionals (psychologists or counselors) |  |  |  |  |  |
| 1. I think seeking mental health help is a mature behavior |  |  |  |  |  |
| 1. I am confident that mental health professionals can effectively help patients |  |  |  |  |  |

**Depression Anxiety Stress Scales (DASS-21)（Single-choice questions）**

|  | Never | Sometimes | Often | Always |
| --- | --- | --- | --- | --- |
| 1. I find it difficult to feel happy or satisfied with anything |  |  |  |  |
| 1. I feel that there is nothing to look forward to in life |  |  |  |  |
| 1. I cannot truly feel happiness, even in things I enjoy |  |  |  |  |
| 1. I feel sad and depressed |  |  |  |  |
| 1. I have lost interest in most things in life |  |  |  |  |
| 1. I feel worthless |  |  |  |  |
| 1. I feel that life has no meaning |  |  |  |  |
| 1. I feel dry in my mouth |  |  |  |  |
| 1. I feel short of breath (even without physical activity) |  |  |  |  |
| 1. I feel anxious and afraid that something bad will happen |  |  |  |  |
| 1. I feel very nervous |  |  |  |  |
| 1. I find myself easily panicking |  |  |  |  |
| 1. I feel scared and extremely anxious |  |  |  |  |
| 1. I find myself easily irritated or impatient |  |  |  |  |
| 1. I feel overly sensitive or prone to anger |  |  |  |  |
| 1. I feel extremely tense or unable to relax |  |  |  |  |
| 1. I find myself feeling frustrated over small things |  |  |  |  |
| 1. I feel I cannot control my worries |  |  |  |  |
| 1. I find myself lacking enough patience in difficult situations |  |  |  |  |
| 1. I feel I lack enough energy for anything |  |  |  |  |
| 1. I feel that things are out of my control |  |  |  |  |

**C部分 数字媒介适应**

**数字心理健康素养量表（MeHLS）（23 items）（Single-choice questions）**

|  | Never | Occasionally | Sometimes | Often | Always |
| --- | --- | --- | --- | --- | --- |
| 1. When I feel I have emotional problems, I try to search online for information to understand my emotions. |  |  |  |  |  |
| 1. When I feel I have emotional problems, I try to use online self-assessment tools for an initial evaluation. |  |  |  |  |  |
| 1. When I feel I have emotional problems, I try to follow social media accounts that provide emotional management information. |  |  |  |  |  |
| 1. When I feel I have emotional problems, I try to collect or share emotional management posts on social media. |  |  |  |  |  |
| 1. When I feel I have emotional problems, I try to share my feelings on social media to relieve stress. |  |  |  |  |  |
| 1. When I feel I have emotional problems, I try to use online platforms to track my emotional changes (e.g., writing an online mood diary). |  |  |  |  |  |
| 1. When I feel I have emotional problems, I try to attend online emotional management courses. |  |  |  |  |  |
| 1. When others have emotional problems, I try to suggest they seek help through online platforms. |  |  |  |  |  |
|  | Strongly Disagree | Disagree | Neutral | Agree | Strongly Agree |
| 1. I know some websites or apps for self-emotional management. |  |  |  |  |  |
| 1. I know some keywords that can help me search online for information related to my emotional problems. |  |  |  |  |  |
| 1. I know online discussion forums where I can gain emotional management experience and community support. |  |  |  |  |  |
| 1. I know how to find online or offline mental health service information that I need. |  |  |  |  |  |
| 1. I know how to find information online to prevent emotional problems. |  |  |  |  |  |
| 1. I know how to find online information for screening or evaluating emotional problems. |  |  |  |  |  |
| 1. I know how to find treatment options for emotional problems online (e.g., traditional therapies or new treatments). |  |  |  |  |  |
| 1. I can select online mental health services that suit my personal needs. |  |  |  |  |  |
| 1. I can assess the quality of online information that helps with my emotional management. |  |  |  |  |  |
| 1. I can identify and avoid certain online information that may trigger negative emotions or stress. |  |  |  |  |  |
| 1. I can manage potential risks associated with using online platforms (e.g., personal data leakage) that cause anxiety. |  |  |  |  |  |
| 1. I can use online platforms to monitor my emotional state and make corresponding changes (e.g., behavior/emotional adjustments). |  |  |  |  |  |
| 1. I can use the knowledge gained from online platforms to improve my emotional awareness. |  |  |  |  |  |
| 1. I can use the knowledge gained from online platforms to enhance my ability to express emotions. |  |  |  |  |  |
| 1. I can use the emotional management methods learned from online platforms to handle similar emotional problems in the future. |  |  |  |  |  |

**Bergen社交媒体成瘾量表（6 items）（Single-choice questions）**

| Over the past year, have you frequently... | Never | Occasionally | Sometimes | Often | Always |
| --- | --- | --- | --- | --- | --- |
| 1. Spent a lot of time thinking about social media or planning to use it？ |  |  |  |  |  |
| 1. Felt an increasing need to use social media? |  |  |  |  |  |
| 1. Used social media to escape worries or stress? |  |  |  |  |  |
| 1. Tried to reduce social media use but were unsuccessful? |  |  |  |  |  |
| 1. Felt irritated or distressed when limited in using social media? |  |  |  |  |  |
| 1. Had negative effects on work or studies due to excessive social media use? |  |  |  |  |  |

**eHealth Literacy Scale (eHEALS)（8 items）（Single-choice questions）**

|  | Never | Occasionally | Sometimes | Often | Always |
| --- | --- | --- | --- | --- | --- |
| 1. I know how to find useful health information online. |  |  |  |  |  |
| 1. I know how to answer my health questions using the internet. |  |  |  |  |  |
| 1. I know what health information resources are available on the internet. |  |  |  |  |  |
| 1. I know where to find reliable health information online. |  |  |  |  |  |
| 1. I know how to use health information found online to help me. |  |  |  |  |  |
| 1. I have the ability to assess the quality of online health information. |  |  |  |  |  |
| 1. I can distinguish between high-quality and low-quality health information online. |  |  |  |  |  |
| 1. I am confident in using online information to make health decisions. |  |  |  |  |  |

Thank you again for your participation. We wish you all the best in your studies and life!
