# Supplementary material for: The Interactive Effects of eHealth Literacy and Mental Health Literacy on Social Media Addiction and Depression-Anxiety-Stress in Adolescents: Cross-Sectional Study
Source: J Med Internet Res. 2025 Nov 28;27:e81741. doi: 10.2196/81741 (PMC12701345; doi:10.2196/81741)
Supplement: Multimedia Appendix 2 [file jmir_v27i1e81741_app2.pdf]

STROBE Statement—Checklist of items that should be included in reports of ***cross-sectional studies***

	Item No	Recommendation	Page No
**Title and abstract**	1	(*a*) Indicate the study's design with a commonly used term in the title or the abstract	I、II
		(*b*) Provide in the abstract an informative and balanced summary of what was done and what was found	I、II
**Introduction**			
Background/rationale	2	Explain the scientific background and rationale for the investigation being reported	1-2
Objectives	3	State specific objectives, including any prespecified hypotheses	8-9
**Methods**			
Study design	4	Present key elements of study design early in the paper	9
Setting	5	Describe the setting, locations, and relevant dates, including periods of recruitment, exposure, follow-up, and data collection	9-15
Participants	6	(*a*) Give the eligibility criteria, and the sources and methods of selection of participants	9
Variables	7	Clearly define all outcomes, exposures, predictors, potential confounders, and effect modifiers. Give diagnostic criteria, if applicable	12-13
Data sources/ measurement	8*	For each variable of interest, give sources of data and details of methods of assessment (measurement). Describe comparability of assessment methods if there is more than one group	*10-12*
Bias	9	Describe any efforts to address potential sources of bias	16
Study size	10	Explain how the study size was arrived at	14-15
Quantitative variables	11	Explain how quantitative variables were handled in the analyses. If applicable, describe which groupings were chosen and why	21-23
Statistical methods	12	(*a*) Describe all statistical methods, including those used to control for confounding	14-15
		(*b*) Describe any methods used to examine subgroups and interactions	21-25
		(*c*) Explain how missing data were addressed	14
		(*d*) If applicable, describe analytical methods taking account of sampling strategy	14
		(*e*) Describe any sensitivity analyses	-
**Results**			
Participants	13*	(a) Report numbers of individuals at each stage of study—eg numbers potentially eligible, examined for eligibility, confirmed eligible, included in the study, completing follow-up, and analysed	14
		(b) Give reasons for non-participation at each stage	14
		(c) Consider use of a flow diagram	-
Descriptive data	14*	(a) Give characteristics of study participants (eg demographic, clinical, social) and information on exposures and potential confounders	17
		(b) Indicate number of participants with missing data for each variable of interest	14
Outcome data	15*	Report numbers of outcome events or summary measures	17
Main results	16	(*a*) Give unadjusted estimates and, if applicable, confounder-adjusted	18-

		estimates and their precision (eg, 95% confidence interval). Make clear which confounders were adjusted for and why they were included	25
		(*b*) Report category boundaries when continuous variables were categorized	24-25
		(*c*) If relevant, consider translating estimates of relative risk into absolute risk for a meaningful time period	-
Other analyses	17	Report other analyses done—eg analyses of subgroups and interactions, and sensitivity analyses	16-25
**Discussion**			
Key results	18	Summarise key results with reference to study objectives	26-28
Limitations	19	Discuss limitations of the study, taking into account sources of potential bias or imprecision. Discuss both direction and magnitude of any potential bias	28
Interpretation	20	Give a cautious overall interpretation of results considering objectives, limitations, multiplicity of analyses, results from similar studies, and other relevant evidence	26-28
Generalisability	21	Discuss the generalisability (external validity) of the study results	28
**Other information**			
Funding	22	Give the source of funding and the role of the funders for the present study and, if applicable, for the original study on which the present article is based	30

*Give information separately for exposed and unexposed groups.

Note: An Explanation and Elaboration article discusses each checklist item and gives methodological background and published examples of transparent reporting. The STROBE checklist is best used in conjunction with this article (freely available on the Web sites of PLoS Medicine at http://www.plosmedicine.org/, Annals of Internal Medicine at http://www.annals.org/, and Epidemiology at http://www.epidem.com/). Information on the STROBE Initiative is available at www.strobe-statement.org.

Title and abstract

Item No 1

(a) Indicate the study's design with a commonly used term in the title or the abstract

Fulfilled. The phrase "A Cross-Sectional Study" is included in the manuscript title.

(b) Provide in the abstract an informative and balanced summary of what was done and what was found

Fulfilled. The abstract provides a structured summary covering Background, Methods (design, participants, measures, analysis), Results (key direct, indirect, and interaction effects with estimates and 95% confidence intervals), and Conclusions, offering a balanced and quantitative overview.

Introduction

Item No 2

Explain the scientific background and rationale for the investigation being reported

Fulfilled. The introduction details the adolescent mental health crisis in China, high social media penetration, relevant national policies, gaps in existing literature (e.g., lack of focus on eHL-MHL interaction, underexplored MeHL), and a theoretical rationale integrating COR theory, HBM, and SCT, providing comprehensive scientific background.

Item No 3

State specific objectives, including any prespecified hypotheses

Fulfilled. Specific objectives and three prespecified hypotheses (H1, H2, H3) are explicitly stated in the "The Current Study" subsection and illustrated in Fig. 1.

Methods

Item No 4

Present key elements of study design early in the paper

Fulfilled. The cross-sectional design is stated in the first sentence of the "Material and Methods" section.

Item No 5

Describe the setting, locations, and relevant dates, including periods of recruitment, exposure, follow-up, and data collection

Fulfilled. The setting (general high schools), locations (five Chinese provinces: Beijing, Zhejiang, Shanxi, Henan, Jiangsu), and dates for data collection (February-April 2025) are described in the "Participants" and "Procedure" subsections. Recruitment and data collection periods coincide.

Item No 6

(a) Give the eligibility criteria, and the sources and methods of selection of participants

Fulfilled. The target population (school-attending adolescents), exclusion criteria (severe physical or mental disabilities, inability to complete questionnaire independently, ongoing related treatment), and detailed selection method (stratified cluster random sampling across provinces, schools, and classrooms) are provided in the "Participants" subsection.

Item No 7

Clearly define all outcomes, exposures, predictors, potential confounders, and effect modifiers. Give diagnostic criteria, if applicable

Fulfilled. Outcomes (SMA, DASS), exposures/predictors (eHL, MHL), the mediator (MeHL), and covariates (age, gender) are clearly defined in a dedicated paragraph in the "Measures" section. MHL is explicitly tested as an effect modifier in Hypothesis 3. All are measured using established scales.

Item No 8

For each variable of interest, give sources of data and details of methods of assessment (measurement). Describe comparability of assessment methods if there is more than one group

Fulfilled. For each variable, the source of the scale (original developer and citation, with adaptation details for MHLS-SF), number of items, dimensions, scoring method (Likert scale details, clarification on DASS-21 scoring), reliability (Cronbach's α, and now McDonald's ω), and validity (CFA results including SRMR in Table 1) are detailed in the "Measures" subsection. All participants received identical assessments.

Item No 9

Describe any efforts to address potential sources of bias

Fulfilled. Procedural controls (anonymity, item randomization) and a statistical test (Harman's single-factor test) for Common Method Bias were employed and reported in the "Common Method Bias Test" subsection.

Item No 10

Explain how the study size was arrived at

Fulfilled. A post hoc power analysis (G*Power) is reported in the "Procedure and Data Analysis" subsection, indicating the sample size (N=855) was sufficient to detect medium/small effects with 95% power.

Item No 11

Explain how quantitative variables were handled in the analyses. If applicable, describe which groupings were chosen and why

Fulfilled. Variables were treated as continuous in primary analyses (SEM, correlation). For moderation analysis, Mental Health Literacy (MHL) was centered and used in its continuous form

for the interaction term. For simple slope analysis and visualization, MHL was grouped into low/medium/high levels, with the reason stated.

Item No 12

(a) Describe all statistical methods, including those used to control for confounding

Fulfilled. All statistical methods (SPSS for descriptives/correlations/CMB; AMOS for CFA/SEM; PROCESS Macro for mediation/moderation) are described. Control for confounding by age and gender in SEM and PROCESS models is explicitly stated. The use of cluster-robust standard errors is now highlighted.

(b) Describe any methods used to examine subgroups and interactions

Fulfilled. The examination of the interaction (eHL × MHL) using hierarchical regression with cluster-robust SEs and the test for moderated mediation (Hypothesis 3) using PROCESS Model 7 with the Johnson-Neyman technique are described.

(c) Explain how missing data were addressed

Fulfilled. The handling of missing data is described in the "Procedure" section: questionnaires with any missing items were excluded, and the number (n=28) and reasons are given.

(d) If applicable, describe analytical methods taking account of sampling strategy

Fulfilled. The stratified cluster sampling strategy is described. The "Procedure and Data Analysis" subsection now explicitly states that all primary analyses (SEM, moderated mediation) were conducted using cluster-robust standard errors with the classroom as the cluster unit to account for the design effect. Intraclass Correlation Coefficients (ICCs) for the outcomes are reported.

(e) Describe any sensitivity analyses

Not Fulfilled. Sensitivity analyses are not mentioned or reported. This can be noted as a limitation in the discussion.

Results

Item No 13

(a) Report numbers of individuals at each stage of study—eg numbers potentially eligible, examined for eligibility, confirmed eligible, included in the study, completing follow-up, and analysed

Fulfilled. Numbers are reported for questionnaires distributed (n=1000), returned (n=893), excluded due to invalidity (n=38), and analyzed (n=855) in the "Procedure" section.

(b) Give reasons for non-participation at each stage

Fulfilled. Reasons for non-participation (student absences for non-returns; incompleteness, abnormal completion time, patterned responses for exclusions) are provided in the "Procedure" section.

(c) Consider use of a flow diagram

Not Fulfilled. A participant flow diagram was not included. While not strictly mandatory, its absence is noted.

Item No 14

(a) Give characteristics of study participants (eg demographic, clinical, social) and information on exposures and potential confounders

Fulfilled. Table 2 provides descriptive statistics (means, standard deviations, correlations) for all variables, including demographics (age, gender), exposures (eHL, MHL), the mediator (MeHL), outcomes (SMA, DASS), and covariates (age, gender).

(b) Indicate number of participants with missing data for each variable of interest

Fulfilled. By stating that questionnaires with any missing items were excluded, it is indicated that the final analyzed sample (n=855) had complete data for all variables of interest.

Item No 15

Report numbers of outcome events or summary measures

FFulfilled. Summary measures (Means, Standard Deviations) for outcome variables (SMA, DASS) and all other variables are reported in Table 2.

Item No 16

(a) Give unadjusted estimates and, if applicable, confounder-adjusted estimates and their precision (eg, 95% confidence interval). Make clear which confounders were adjusted for and why they were included

Fulfilled. Confounder-adjusted estimates (standardized path coefficients β, with cluster-robust Bootstrap confidence intervals) from SEM and PROCESS analyses, which controlled for age and gender, are reported in Tables 3, 4, 5, and 6. The confounders and rationale for inclusion are stated.

(b) Report category boundaries when continuous variables were categorized

Fulfilled. The categorization of the continuous variable MHL for the simple slope analysis was based on 16th、50th、84th, as implied by the methodology and figure.

(c) If relevant, consider translating estimates of relative risk into absolute risk for a meaningful time period

Not Applicable. The study reports associative measures (path coefficients, correlations) and not relative risk estimates.

Item No 17

Report other analyses done—eg analyses of subgroups and interactions, and sensitivity analyses

Fulfilled. Other analyses, including common method bias test, descriptive statistics, correlation analysis, confirmatory factor analysis, mediation, moderation, and moderated mediation analyses, are reported.

Discussion

Item No 18

Summarise key results with reference to study objectives

Fulfilled. The first paragraph of the discussion quantitatively summarizes key results with direct reference to the testing of the pre-specified hypotheses (H1, H2, H3), including effect estimates and confidence intervals.

Item No 19

Discuss limitations of the study, taking into account sources of potential bias or imprecision. Discuss both direction and magnitude of any potential bias

Fulfilled. The "Limitations" subsection discusses the cross-sectional design (causality and reverse causality), self-report data (social desirability bias, lack of objective validation), limited generalizability (regional sample, lack of key demographics like SES), unmeasured confounders, and the lack of sensitivity analysis. The potential direction of bias is addressed.

Item No 20

Give a cautious overall interpretation of results considering objectives, limitations, multiplicity of analyses, results from similar studies, and other relevant evidence

Fulfilled. The discussion provides a cautious interpretation by integrating results with objectives, acknowledging limitations, using associative language, linking findings to relevant theories (COR, SCT, HBM) and previous studies, and positioning the study as a foundation for future longitudinal research.

Item No 21

Discuss the generalisability (external validity) of the study results

Fulfilled. The generalizability is explicitly discussed in the "Limitations" section, noting the regional sampling frame within China and the lack of data on urban/rural residence, ethnicity, and SES. The conclusions are framed cautiously regarding national application.

Other information

Item No 22

Give the source of funding and the role of the funders for the present study and, if applicable, for the original study on which the present article is based

Fulfilled. The "Funding" section declares the source (National Social Science Fund of China) and the role of the funder ("involved in the study design and provided supervision and mentorship throughout the manuscript preparation process").
